# Enhanced bacterial cellulose production by *Komagataeibacter xylinus* using agro-derived flour nitrogen sources

**DOI:** 10.1007/s00253-025-13702-z

**Published:** 2026-01-29

**Authors:** Dheanda Absharina, Csilla Veres, Alfonz Kedves, Zoltán Kónya, Balázs P. Szabó, Csaba Vágvölgyi

**Affiliations:** 1 Department of Biotechnology and Microbiology, Faculty of Science and Informatics, University of Szeged, Közép Fasor, 6726 Szeged, Hungary; 2 Institute of Animal Science and Wildlife Management, Faculty of Agriculture, University of Szeged, Hódmezővásárhely, Hungary; 3 Department of Applied and Environmental Chemistry, University of Szeged, Szeged, Hungary; 4 HUN-REN-SZTE Reaction Kinetics and Surface Chemistry Research Group, Szeged, Hungary; 5 Institute of Food Engineering, Faculty of Engineering, University of Szeged, Szeged, Hungary

**Keywords:** *Komagataeibacter xylinus*, Flour-based substrates, Agricultural waste valorization, Nitrogen substitutions, Crystallinity

## Abstract

**Abstract:**

Bacterial cellulose (BC) production is limited by the high cost of refined nitrogen sources such as yeast extract and tryptone. While carbon source substitution has been widely studied, approaches for nitrogen replacement remain underexplored. Here, we evaluated cereal and pseudo-cereal flours as low-cost nitrogen alternatives for *Komagataeibacter xylinus* DSMZ 2325 under static fermentation using two nitrogen substitution strategies: constant total nitrogen (CTN) and constant nitrogen source mass (CNSM). Thirteen flour variants were screened, with maximum BC yields of 3.40 g/L (soy), 3.17 g/L (teff), 2.74 g/L (quinoa), and 1.94 g/L (triticale) under the CTN strategy, in several cases surpassing the modified HS control. Structural analyses confirmed that flour-derived BC retained the defining characteristics of high-quality cellulose, including high crystallinity, robust fibrillar networks, and thermal stability. Soy consistently produced the highest volumetric yields, whereas triticale exhibited the greatest fold-increase compared to the control, highlighting the distinct nutritional and compositional contributions. These results demonstrate that flour-based media provide a viable strategy to reduce production costs while tailoring BC properties through substrate choice. By shifting the focus from carbon to nitrogen optimization, this study introduces a sustainable and scalable approach to bioprocessing using agro-derived raw materials.

**Key Points:**

*• Flour-based nitrogen sources replace costly yeast extract and tryptone in BC media*

*• Triticale, teff, quinoa, and soy influence BC yield and cellulose fibril formation*

*• Agro-flour substrates offer low-cost, sustainable bacterial cellulose production*

## Introduction

Bacterial cellulose (BC) is a nanofibrillar biopolymer synthesized predominantly by *Komagataeibacter* spp., valued for its high purity (~ 97%), mechanical strength, and exceptional water-holding capacity, which enable applications in wound dressings, food technology, packaging, and textiles (Lahiri et al. 2021; Esa et al. 2014; Khazeni et al. 2017). BC is also applied in acoustic devices, medical dressings, and specialty products (Lee et al. 2015), with promising roles in artificial blood vessels, biodegradable scaffolds, flexible electronics, and sensors (Hu et al. 2014). Furthermore, BC has attracted attention as a sustainable leather substitute due to its moldability, biocompatibility, > 90% water retention, and tensile strength more than tenfold higher than plant cellulose (Garcia and Prieto 2019).

Despite its broad applicability in biomedical, food, and material science sectors, large-scale BC production remains limited by the high cost of glucose-based synthetic media, with culture medium expenses representing ~ 30% of total production costs (Ul-Islam et al. 2017; Hong and Qiu 2008; Fernandes et al. 2020; Revin et al. 2018). To address this, numerous studies have explored low-cost or waste-derived carbon substrates, including fruit juices, agro-industrial residues, and lignocellulosic hydrolysates. For instance, muskmelon (8.08 g/L), orange (6.18 g/L), and watermelon (5.98 g/L) juices (Wang et al. 2018), along with coconut and pineapple juices (Kurosumi et al. 2009), produced BC yields comparable to glucose media. Sugarcane molasses (39% sugars) achieved up to 76% yield after acid–heat pretreatment, while pretreated cotton textile waste (10.8 g/L; Costa et al. 2017), confectionery waste hydrolysates (13.0 g/L; Tsouko et al. 2015), and thin stillage (10.38 g/L; Wu and Liu 2012) also yielded competitive results.

Nitrogen replacement strategies have likewise enhanced cost-efficiency. Protein-rich residues such as rice bran have successfully substituted yeast extract and peptone, improving yields by up to 2.4-fold (Narh et al. 2018; Sutanto et al. 2017). Tea infusions, particularly black and green, are effective in composite cultures such as SCOBY and *Medusomyces gisevii* Sa-12 (Yim et al. 2017; Sharma and Bhardwaj 2019), while other nitrogen-rich alternatives include corn steep liquor/powder (Quijano et al. 2024), soybean molasses (2.23–15.68 g/L; Souza et al. 2020), tofu-processing residues (4.14–5.52 g/L; Suwanposri et al. 2014), and cashew-derived wastes (Souza et al. 2020). More recently, Henry et al. (2024) demonstrated that solid-state fermentation of cereal waste generates nutrient-rich hydrolysates, underscoring the potential of integrated carbon–nitrogen valorization.

Using agro-industrial byproducts not only reduce BC production costs but also mitigate environmental impacts and sustainability through waste valorization (Souza et al. 2020). Nonetheless, some feedstocks require pretreatment to remove inhibitory compounds. For example, yield improvements have been demonstrated by detoxifying crude glycerol (Soemphol et al. 2018), steam-distilling tobacco waste to eliminate nicotine (Ye et al. 2019) and applying a two-step process to waste beer yeast (Lin et al. 2014). Similarly, the citrus peel and pomace hydrolysate obtained by enzymatic treatment (CPPE medium) yielded 5.7 ± 0.7 g/L BC, substantially higher than the HS medium (Fan et al. 2016). Thus, selecting low-inhibitor substrates that require minimal or targeted pretreatment is essential for cost-effective, high-yield BC production. Because nitrogen availability strongly influences BC synthesis, the nitrogen profile of agro-flours is essential in determining their suitability for medium reformulation.

Although many studies have focused on carbon-source substitution, the systematic replacement of refined nitrogen sources remains considerably underexplored. Most agro-waste substrates evaluated to date are carbon-rich and compositionally heterogeneous, limiting their usefulness for mechanistic assessment of nitrogen effects on BC biosynthesis. In contrast, cereal and pseudo-cereal flours represent a distinct class of agro-derived materials with reproducible nitrogen content, defined protein quality, and consistent amino acid profiles. This work provides the first comprehensive assessment of how nitrogen-rich flours can simultaneously reduce medium costs and modulate BC yields and structural properties.

Accordingly, this study evaluated cereal- and legume-based flours derived from rice, corn, wheat, soy, oats, spelt, quinoa, millet, triticale, teff, bulgur, rye and sorghum as alternative fermentation media for *K. xylinus* DSMZ 2325, leveraging their nitrogen content to enhance BC productivity under constant total nitrogen (CTN) content (0.6 g) and constant nitrogen source mass (CNSM) regime (5 g). The resulting pellicles were characterized by Fourier transform infrared spectroscopy (FTIR), scanning electron microscopy (SEM), and X-ray diffraction (XRD) to assess structural and crystallinity properties. To our knowledge, this represents the first systematic evaluation of agro-derived nitrogen supplementation for BC production, offering a sustainable approach to biomanufacturing using agro-industrial byproducts.

## Methodology

### Strain maintenance and inoculum preparation

*Komagataeibacter xylinus* DSMZ 2325 was maintained on modified Hestrin-Schramm (HS) agar at 4 °C to ensure viability. For inoculum preparation, three loopfuls from a seven-day-old culture were aseptically transferred into 50 mL of HS broth and incubated at 28 °C with agitation at 120 rpm for 48 h. The culture was grown until an optical density at 600 nm (OD₆₀₀) of 0.7–1.0 was achieved prior to inoculation into fermentation experiments, consistent with standard practice for *Komagataeibacter* inoculum preparation (Reiniati et al. 2017). The isolated pure cultures were deposited in the Szeged Microbiology Collection (SZMC, http://szmc.hu), Department of Biotechnology and Microbiology, University of Szeged, Hungary.

### Nitrogen content analysis of flour variant

Thirteen pulse, cereal, and pseudo-cereal variants (soy, corn, wheat, oat, rice, bulgur, millet, teff, triticale, quinoa, spelt, rye, and sorghum) were commercially obtained. Grains and flakes were ground (BH/9153; Budapest, Hungary), while flour samples were used directly. Approximately 1.0 g of each sample was oven-dried at 130 °C for 1 h to constant mass (Binder ED115) and reweighed.

Moisture content (N, % m/m) was calculated as:

$$\mathrm{N }= \mathrm{ } \left(\frac{\mathrm{d}}{\mathrm{b}}\right)\times{100}$$

where d is the mass loss (g) and b are the initial sample mass (g).

Total nitrogen content (${\omega }_{N}$, %) was determined by the Kjeldahl method using a Kjeltec™ 2300 automatic distillation–titration unit (FOSS, Hillerød, Denmark) and with standardized H_2_SO_4_ titration, following ISO 16634–2.

Crude protein (${\omega }_{p}$, %) was calculated as:

$${}\omega_{\mathrm{p}}\left(\mathrm{\%}\right)\mathrm{=}{}\omega_{\mathrm{N}}\times{\mathrm{F}}$$

where F is the factor specific to each cereal, pulse, or pseudo-cereal product (as listed on Supplementation Table 1). Crude protein as dry matter (${\omega }_{pd}$, %) was estimated from the total nitrogen content using the appropriate nitrogen-to-protein conversion factor (F), according to ISO 16634–2:2016. The general formula applied was:

$${\omega }_{pd} = \frac{100 {\omega }_{p}}{100- {\omega }_{H20}}$$

**Table 1:** Nitrogen and protein content of agro-derived flour variants determined by the Kjeldahl method. Nitrogen (%) values are presented first, followed by calculated protein (%) values using the appropriate conversion factors

No	Flour Variant	Nitrogen % (Kjedahl)*	Nitrogen % (ref.)	Protein % (Kjedahl)*	Protein % (ref.)
1	Soy	7.22%	6.90%1,3	44.52%	38.6%; 50.00%1,3
2	Corn	1.08%	1.40%1,3	7.39%	7.56%1,3
3	Wheat	1.75%	2.01%1,3	10.79%	9–14%1,3
4	Rice	1.17%	1.11%1,3	7.83%	6.91%1,3
5	Oat	1.28%	1.90%1,3	8.44%	12.50%;13.50%1,3
6	Bulgur	1.81%	1.88%1,3	11.82%	12.32%1,3
7	Millet	1.94%	1.85%1,2,3	12.25%	10.75%1,2^,3^
8	Spelt	2.62%	2.32%1,3^,4^	16.67%	14.50% 16.60–20.00%1,3,^4^
9	Quinoa	2.09%	2.04%1,7	13.15%	13.50%1,7
10	Triticale	1.72%	2.25%1,3^,6^	11.11%	9.80–13.90%1,3,6
11	Teff	1.20%	2.13%1,3^,5^	8.47%	9.07%; 10.21–13.30%1,3,5
12	Rye	1.58%	1.44%1,3	10.01%	6.50–9.00% 8.40%1,3
13	Sorghum	1.24%	1.42%1,2,3	7.78%	7.70%1,2,3

^*^Based on Supplementary Table 1 determined by Kjeldahl analysis with protein content calculated using the appropriate nitrogen-to-protein conversion factor

### Media formulation and flour variant preparation

The control medium for *K. xylinus* DSMZ 2325 cultivation used in all comparative experiments was a modified HS medium, containing (g/L): sucrose 80.0, yeast extract 2.5, tryptone 2.5, acetic acid 6.25, and sodium hydroxide 2.8. In experimental treatments, yeast extract and tryptone were completely substituted with flour variants, without additional pH adjustment, including the HS modified control media. The composition of the control and flour-based media are summarized in *Supplementary Table S2.*

Two nitrogen-replacement regimes were evaluated. In both strategies, yeast extract and tryptone were entirely omitted, and nitrogen was supplied exclusively by the flour.

1. Constant Total Nitrogen (CTN) regime – In this regime, the total nitrogen concentration of the medium was fixed at 0.6 g N/L, equivalent to the nitrogen supplied by the control media, with full (100%) substitution of yeast extract and tryptone. The required mass of each flour was calculated based on its Kjeldahl nitrogen content, which corresponded closely to values reported in the literature, with minor expected variations, ensuring that each CTN medium contained exactly 0.60 g N/L.
2. Constant Nitrogen Source Mass (CNSM) regime – In this regime, each flour was added at a fixed mass of 5.00 g/L. As in the CTN regime, yeast extract and tryptone were fully omitted, making flour the sole nitrogen source. Thus, the resulting nitrogen concentration varied among flours.

A schematic summarizing of the control medium and both nitrogen-replacement strategies has been added as Supplementary Figure S1 to ensure complete clarity regarding media composition and experimental setup.

### Growth kinetics

50 mL flasks of the growth medium were inoculated and incubated at 28 °C, 120 rpm. Samples taken at 0, 6, 24, 30, 48, 54, 72, and 76 h were serially diluted, and 100 µL from serial dilutions ≥ 10^4^ were spread onto modified HS agar. Plates were incubated at 28 °C for 5–7 days, and colonies were enumerated using the total plate count method. Growth curves were generated by plotting log CFU/ml against time to determine the optimal inoculation point for static fermentation.

### Fermentation procedure

Static fermentations were performed in sterile 250 mL glass jars (Gastro preserving jars, product no. 222202011) which fitted with modified cotton-plugged lids, containing a total working volume of 50 mL (45 mL culture medium and 5 mL inoculum; 10% v/v), corresponding to approximately 20% vessel filling and providing sufficient headspace for an effective air–liquid interface (Hornung et al. 2006; Hsieh et al. 2016; Rodrigues et al. 2019; Kumar et al. 2021; Loh et al. 2025). Cultures were incubated at 28 °C for 7 days under static conditions, after which BC pellicles formed at the air–liquid interface were harvested, rinsed with distilled water, and processed for further analyses, consistent with established static BC cultivation protocols (Kumar et al. 2021; Loh et al. 2025). Fermentation broth pH was measured at the start (pH₀) and end (pH₁) of cultivation in triplicate using pH indicator strips (Macherey–Nagel, pH 4.0–7.0).

### Purification and dry weight determination

BC pellicles were harvested and purified by alkaline treatment (5 M NaOH, 100 °C, 30 min), followed by repeated washing with distilled water until neutral pH was reached (pH 7.0). Purified pellicles were then oven-dried at 120 °C to constant mass, and BC yield was determined as dry mass per culture volume (g/L). Comparable NaOH-based purification, washing-to-neutrality, and dry-weight yield determination procedures have been widely used in recent BC studies (Carreira et al. 2011; Wu et al. 2019; Volova et al. 2022).

### Physicochemical and morphological characterization

Prior to analysis, BC pellicles were lyophilized at –40 °C and 800 mbar for 24 h in a vacuum drying unit (ALPHA 1–4/LSCplus, Martin Christ Drying Systems GmbH, Osterode, Germany). For morphological assessment, a subset of pellicles was selected based on their representative performance profiles, as determined by fold-change analysis relative to the HS control. These highest-performing pellicles were subsequently used for detailed physicochemical and SEM characterization.

### Scanning Electron Microscopy (SEM)

Freeze-dried BC pellicles were cut into small sections and sputter-coated with gold and mounted on slides using conductive carbon type. Samples were examined under SEM (Hitachi S-4700 Type II; Hitachi, Tokyo, Japan) at 10 kV (Kedves et al. 2021). BC fibril diameters were measured from SEM micrographs using ImageJ (v1.54) with the DiameterJ plugin (https://www.nist.gov/mml/bbd/biomaterials/diameterj; Hotaling et al. 2015). For each micrograph, measurements were collected from 50 distinct sites, yielding approximately 5,000–20,000 individual fibril diameter values from randomly selected, non-overlapping regions. These data were used to construct fibril diameter distribution histograms for each BC sample.

### X-ray Diffraction (XRD)

Thin films of BC were prepared on glass slides. Crystalline structure was analyzed using an X-ray diffractometer (D8 Advance, Bruker, Germany) with Cu Kα radiation source (λ = 0.1541 nm) at 40 kV and 20 mA. Diffraction patterns were collected over 2θ = 10°–50° at 1° s⁻^1^. The results of diffractograms subsequently were analyzed using Origin 2023 Peak Analyser software. Crystallinity index (CrI %) was calculated by the Segal method (Segal et al. 1959; Henry et al. 2024):

$$\text{Crystallinity Index }\left(\mathrm{CI}\right)=\frac{{\mathrm{I}}_{200} - {\mathrm{I}}_{\mathrm{am}}}{{\mathrm{I}}_{200}} \times 100$$

where I_200_ is the maximum intensity of the (200) reflection at 22.7° and I_am_ is the minimum intensity around 18°–19°, representing the amorphous fraction. CrI values were derived from representative single measurements per condition and are reported for comparative structural assessment, consistent with prior BC studies (Mohammadkazemi et al. 2015; Dubey et al. 2017).

### Fourier Transform Infrared Spectroscopy (FTIR)

FTIR spectra of dried BC films were recorded (ATR-FTIR, Thermo Scientific, Waltham, MA, USA) over 4500–500 cm⁻^1^, resolution 4–16 cm⁻^1^, 200 scans. Baseline correction and normalization were applied to reduce noise and enable qualitative comparison across reformulated media. Data were processed in Origin 2023, following established protocols for cellulose-based fibers (Qosim et al. 2024; Montoya et al. 2022).

### Thermogravimetric Analysis (TGA)

Thermal stability was analyzed on Mettler Toledo (Melbourne, Australia) instruments. Dried samples (3 mg) were heated from room temperature to 800 °C at 10 °C min⁻^1^ under nitrogen flow (25 mL min⁻^1^). For oxidative stability, samples were further heated in air to 600 °C at 25 °C min⁻^1^. Mass loss (TGA) and derivative curves (DTG) were evaluated (Burhenne et al. 2013).

### Statistical Analysis

All experiments were performed in biological triplicate. Statistical analyses were carried out in GraphPad Prism (v10.2.3) using one-way ANOVA with Tukey’s post hoc test (p < 0.05). SEM fiber diameters were analyzed in RStudio (2022.02.0; RStudio, PBC) to generate histograms and heatmaps. FTIR and XRD data were processed in OriginPro 2023.

## Results

### Kinetic growth curve analysis

**Fig. 1:**
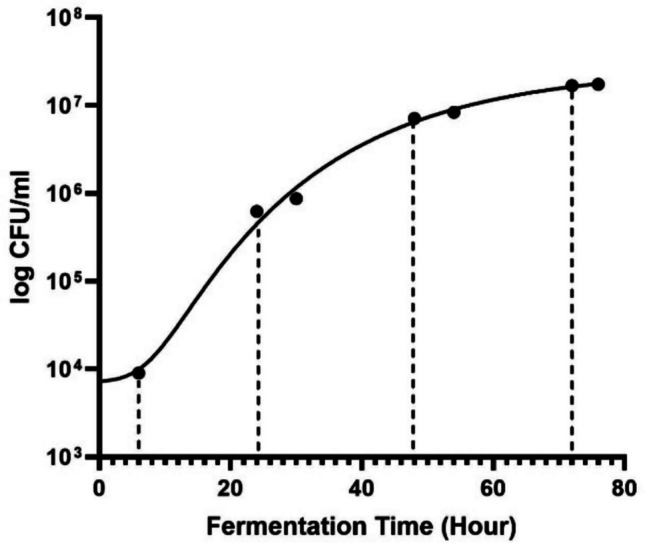
Kinetic growth curve of *Komagataeibacter xylinus* DSMZ 2325 during a 76-h propagation phase under static culture

Understanding the relationship between growth kinetics and BC synthesis is crucial for optimizing yields. In the present study (Fig. 1), *K. xylinus* DSMZ 2325 exhibited well-defined lag, log, and stationary phases, while a death phase was not evident. Growth monitored by over 76 h showed a rapid increase between 24–54 h before entering the stationery and decline phases. A typical bacterial growth curve under batch culture is comprised of four phases: lag, exponential (log), stationary, and death (Rolfe et al. 2012).

The lag phase lasted only about 6 h after inoculation. followed by a rapid increase in viable cell count between 24 and 54 h, corresponding to the exponential phase. Cell density reached approximately 10^7^ CFU/ml and plateaued after 72 h, indicating the transition to stationary phase. The transition from exponential to stationary growth coincided with the onset of BC pellicle formation. The shortened lag phase observed here was likely a result of cultivation under optimized conditions, specifically modified HS medium buffered at a constant pH of 5 and maintained at 28 °C with agitation at 120 rpm.

### Nitrogen and protein content of agriculture-derived flour variants in CTN and CNSM regimes

The control medium for *K. xylinus* DSMZ 2325 cultivation was a modified HS medium containing sucrose, yeast extract, and tryptone. For all flour-based experimental treatments, yeast extract and tryptone were completely omitted, and nitrogen was supplied exclusively by the flour variants. In the CTN regime, the amount of flour added was adjusted to provide a total of 0.6 g nitrogen per liter, whereas in the CNSM regime a fixed flour mass of 5 g/L was used without adjusting for nitrogen content. The complete compositions of the control and flour-based media are summarized in Supplementary Table S2. Nitrogen content of the flour variants was estimated using the Kjeldahl method, which is a standard analytical procedure for determining total protein content. The carbon source (sucrose) was kept constant at 80 g/L in all media formulations.

Kjeldahl analysis (Table 1) revealed substantial variation in nitrogen and protein content among the tested flours. Soy flour contained the highest nitrogen and protein contents (44.52% protein; 7.22% N). Among the cereal flours, spelt (2.93% N; 18.28% protein) and quinoa (2.31% N; 14.42% protein) showed intermediate values, while triticale (1.78% N; 11.11% protein), millet (1.96% N; 12.25% protein), and teff (1.36% N; 8.47% protein) exhibited lower nitrogen and protein contents. Notably, teff supported strong cellulose yields despite its modest protein content, suggesting that amino acid balance and mineral composition can compensate for limited nitrogen supply. In contrast, spelt and millet, although richer in protein, did not produce comparable yields, indicating that protein concentration alone is not reliably predictor of BC productivity. These findings are broadly consistent with reference values for nitrogen and protein contents reported for diverse agro-products, confirming the accuracy of the present dataset on Table 1.

Reference nitrogen and protein values were obtained from USDA Food Data Central [1]; ARS reports [2]; Arendt & Zannini 2013 [3]; Kandić et al. 2023 [4]; Quan et al 2023 [5]; Messina et al 2024 [6]; Ogungbenle, 2003 [7].

### Substrate screening, and BC production performance: influence of nitrogen content and protein quality

BC yields varied significantly with both nitrogen concentration and protein quality of the flour substrates. Soy, with the highest nitrogen level (7.22% N; 44.5% protein), produced 3.40 ± 0.25 g/L at full substitution under CTN conditions and 1.47 ± 0.40 g/L under the nitrogen-limited CNSM regime. Complete yield data for soy and all other flours are provided in *Supplementary Table S3*. Cultures supplemented with quinoa and triticale flours also achieved strong yields, likely owing to their favorable amino acid composition, despite their lower crude nitrogen contents (2.09% and 1.72% N, respectively). In contrast, cereals such as corn, rice, and sorghum (1.1–1.2% N; 7–8% protein) incapable to sustain BC synthesis, confirming nitrogen deficiency as a limiting factor in BC biosynthesis (Molina-Ramírez et al. 2017).

**Fig. 2:**
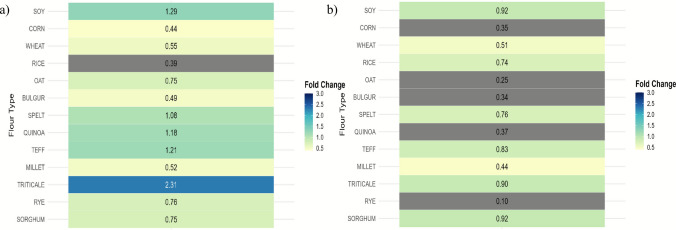
Fold-change heatmap of BC yield under 100% flour substitution relative to the control: (**a**) Constant Total Nitrogen (CTN) content (0.6 g/L); (**b**) Constant Nitrogen Source Mass (CNSM) regime (5 g/L)

Overall screening of the 13 cereal and pseudo-cereal flours therefore revealed pronounced differences in their ability to support BC production. In both experimental strategies, the highest yields were consistently obtained using soy, quinoa, teff, and triticale, each exceeding the modified HS control (Fig. 2).

Under the CTN regime (Fig. 2a), soy, quinoa, teff and triticale consistently produced the highest yields, each exceeding the modified HS control, which were soy (3.40 ± 0.25 g/L), quinoa (2.74 ± 0.15 g/L), teff (3.17 ± 0.30 g/L), and triticale (1.94 ± 0.05 g/L). Triticale and teff showed the strongest fold-change increases (2.31 and 1.21, respectively), followed by quinoa (1.18) and soy (1.29). In contrast, bulgur (1.96 ± 0.02 g/L; fold-change = 0.49), wheat (2.72 ± 0.25 g/L; fold-change = 0.55), corn (1.84 ± 1.00 g/L; fold-change = 0.44), and rye (2.52 ± 0.61 g/L; fold-change = 0.76) produced reduced yields relative to the control.

By comparison, the CNSM regime (Fig. 2b), fold-change analysis showed an overall yield suppression due to nitrogen limitation. Most cereals, including corn, millet, bulgur, and sorghum exhibited fold-change values ≤ 0.5, confirming their limited ability to sustain BC production under low-nitrogen conditions. Soy maintained the highest yield (1.47 ± 0.40 g/L; fold-change = 0.92), reflecting its elevated nitrogen content (7.22%). Comparable relative performances were observed with triticale (0.76 ± 0.06 g/L; fold-change = 0.90), sorghum (0.97 ± 0.09 g/L; fold-change = 0.92), and teff (2.18 ± 0.30 g/L; fold-change = 0.83). In contrast, oats (1.04 ± 0.11 g/L; fold-change = 0.25), bulgur (1.39 ± 0.20 g/L; fold-change = 0.34), quinoa (1.01 ± 0.16 g/L; fold-change = 0.37), and corn (0.60 ± 0.13 g/L; fold-change = 0.35) produced the lowest yields, despite nitrogen contents comparable to triticale, sorghum, and teff. This discrepancy suggests the presence of inhibitory compounds in these substrates.

Besides nitrogen availability, pH regulation also represents another major constraint in *Komagataeibacter* cultivation, as the optimum for *Acetobacter* strains is around 5.5, yet gluconic acid accumulation can reduce the pH to below 3.5 during fermentation (Chawla et al. 2009; Bilgi et al. 2016). As shown in Table 2, initial pH strongly influenced BC yields among the top-performing flours. Although all cultures eventually converged to a final pH near 3.8, differences in the initial pH (pH₀) correlated with yield outcomes.

**Table 2:** Initial (pH₀) and final (pH₁) culture pH and BC yields obtained under the constant total nitrogen (CTN) regime (0.6 g/L), including the BC yield from the modified HS control medium (0% substitution)

Constant Total Nitrogen (CTN) content 0.6 g				
Flour Type	pH_0_	pH_1_	Control (0%; g/L)	Flour Substitution (100%; g/L)
Soy	5.5	3.8	2.63 ± 0.33	3.40 ± 0.25
Quinoa	5.8	3.8	2.33 ± 0.14	2.74 ± 0.15
Teff	5.5	3.8	2.63 ± 0.33	3.17 ± 0.30
Triticale	5.2	3.8	0.84 ± 0.02	1.94 ± 0.05

^*^Control (0%) corresponds to BC–HS modified (g/L), representing the BC yield obtained in the modified Hestrin–Schramm control medium containing sucrose, yeast extract, and tryptone

Soy and teff, both starting at pH₀ 5.5, produced the highest yields (3.40 ± 0.25 and 3.17 ± 0.30 g/L, respectively), indicating that maintaining an optimal starting pH supports stable cellulose production. Quinoa, with a slightly higher pH₀ (5.8), also achieved a strong yield (2.74 ± 0.15 g/L). By contrast, triticale, which began at a lower pH₀ (5.2), showed a reduced absolute yield (1.94 ± 0.05 g/L) despite demonstrating the greatest relative improvement compared to the control. BC–HS modified (g/L) represents the BC yield obtained in the modified Hestrin–Schramm control medium containing sucrose, yeast extract, and tryptone. The full set of BC yield data for all flour variants are provided *in Supplementary Table S3.*

### Structural characterization of BC

#### SEM morphological analysis

**Fig. 3:**
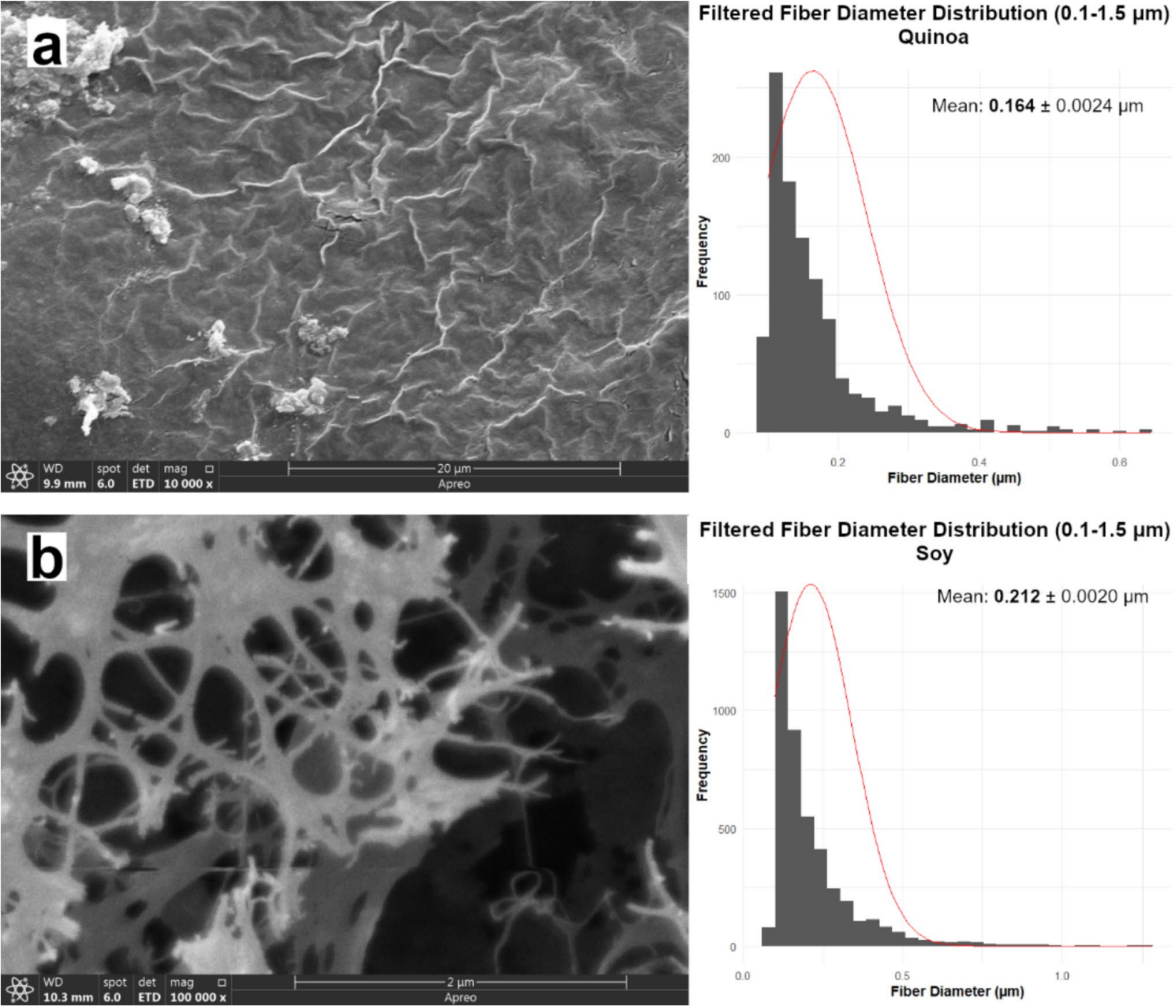

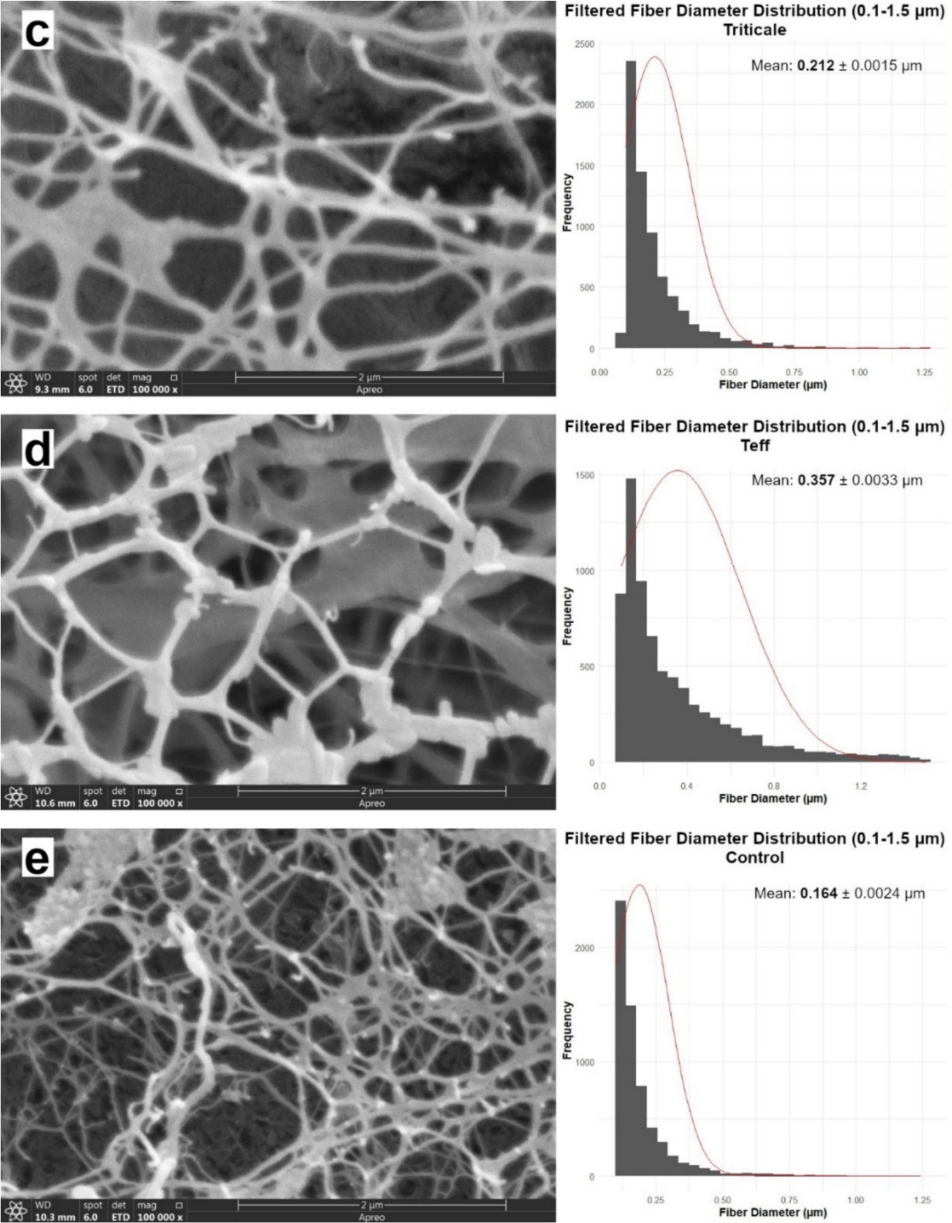
SEM of BC cultivated with different flour-based nitrogen sources, alongside corresponding fiber diameter distribution histograms. Samples include (a) quinoa-derived BC, (b) soy-derived BC, (c) triticale-derived BC, (d) teff-derived BC, and (e) modified HS control BC

Under equal nitrogen input (0.6 g/L), SEM analysis revealed that flour substrates (soy, quinoa, teff, triticale) revealed clear differences in the fibril organization of BC. As shown in Fig. 3, fibril arrangement and network compactness varied notably among samples. Triticale-BC and teff-BC displayed compact and well-integrated nanofibril networks, forming smooth and continuous surfaces. In contrast, quinoa-BC and soy-BC exhibited irregular, disrupted fibrils with nonuniform alignment.

Although individual fibril diameters varied only modestly, the relative abundance of micro- and nanofibrils shifted substantially, leading to distinct nanofibrillar architectures. The SEM imaging collectively demonstrates that even under constant nitrogen supply, substrate identity strongly influences BC ultrastructure, as shown in Fig. 3. Under CTN conditions (0.6 g/L), fibril diameters ranged from 0.164 µm (quinoa-BC) to 0.357 µm (teff-BC), with soy-BC, triticale-BC, and the HS modified control media in the 0.187–0.212 µm range.

Accordingly, the quinoa-derived BC pellicle was imaged at 10,000 ×, representing the highest magnification at which the fibrillar network could be clearly visualized without charging-related artefacts. In contrast, soy-, triticale-, teff-derived, and control BC pellicles exhibited smoother and more homogeneous surfaces, allowing stable imaging at 100,000 × with well-defined nanofibrillar networks. Consistent with previous SEM observations of bacterial cellulose (Lee et al. 2015), lower-to-intermediate magnifications (5,000–10,000 ×) are optimal for visualizing bundled microfibrillar architectures on rough or heterogeneous pellicle surfaces, whereas higher magnifications preferentially resolve nanofibrillar features in compact, homogeneous networks. Collectively, these results indicate that flour-dependent variations in pellicle surface organization directly influence the apparent fibrillar scale observed by SEM.

The diameter distributions derived from SEM image analysis further reflected these structural differences. The histograms adjacent to the SEM imaging indicate that while the triticale pellicle had denser structure and smaller diameter (0.212 µm), the teff pellicle had a less dense structure with larger diameter (0.357 µm). The BC pellicles produced from soy-BC and quinoa-BC exhibited thinner fiber diameter structure and disrupted, disordered organized fibrillar structure despite the high nitrogen content of the flour (7.22% and 2.09%, respectively) compared to the pellicles produced with the low-nitrogen triticale and teff flours (1.72% and 1.20%, respectively) displayed thicker and more ordered nanofibrillar networks.

### FT-IR and XRD analysis

**Fig. 4:**
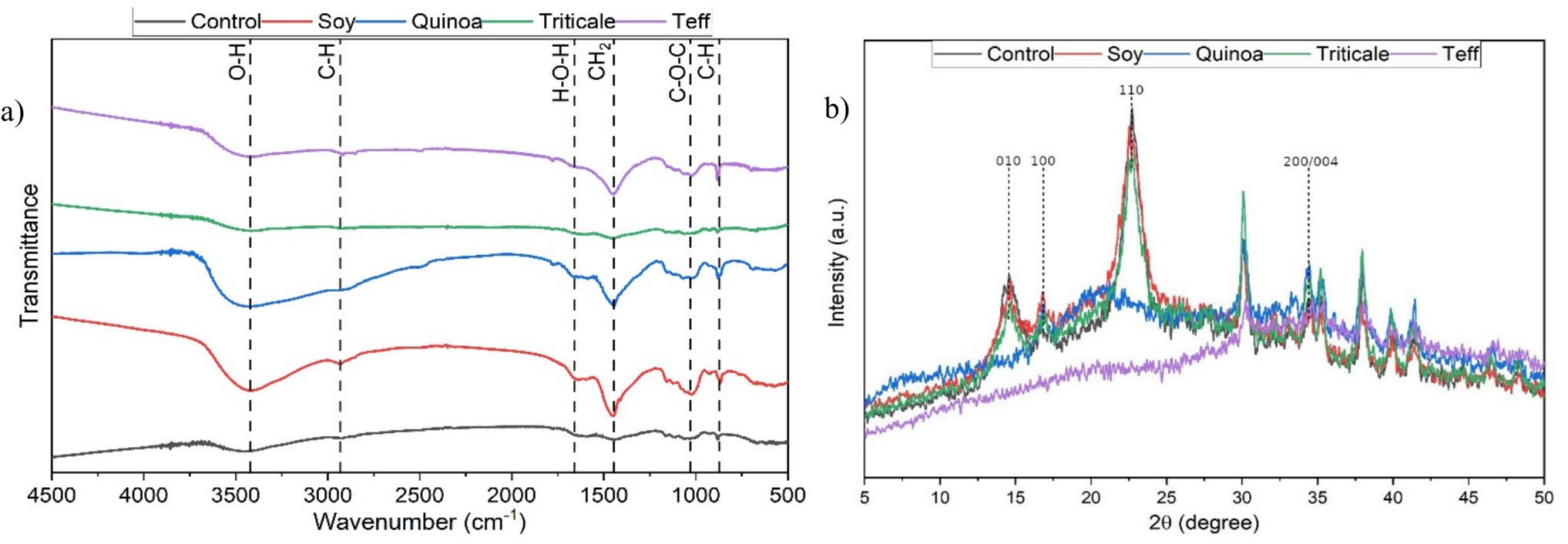
Structural characterization of bacterial cellulose (BC) produced using flour-based nitrogen sources compared with the modified HS control. (a) FTIR spectra showing characteristic polysaccharide absorption bands and substrate-specific differences indicating variations in fibril assembly and residual protein or lipid content. (b) XRD patterns confirming cellulose I structures across all samples, with soy-, quinoa-, triticale-, and teff-derived BC exhibiting distinct differences in crystallinity relative to the control

The ATR–FTIR spectra confirmed the characteristic features of BC, including O–H stretching (3300 cm − 1), C-H stretching (2900 cm − 1), adsorbed water (1640 cm − 1), CH2 bending (crystallinity band, 1427 cm − 1), C-H bending (1350 cm − 1), C–O–C stretching (1160–1110 cm − 1), and C-O stretching (1060 cm − 1). These peaks are consistent with those reported for BC produced from conventional media (Revin et al. 2018; Reiniati et al. 2017; Henry et al. 2024). Strong absorptions at 1160–1030 cm⁻^1^ (C–O–C and C–O stretching) and 895–900 cm⁻^1^ (β-(1 → 4)-glycosidic linkages) confirmed the cellulose I structure, consistent with earlier reports (Irham et al. 2020; Avcioglu et al. 2021; Volova et al. 2022). FTIR analysis of BC produced from 4 different flour variants revealed characteristic cellulose peaks, confirming the preservation of a cellulose type I structure (Fig. 4a). The FTIR and XRD spectra were presented following a format widely adopted in recent studies (Gao et al. 2025; Kedves and Kónya 2025a,b).

The BC samples produced with soy-BC, quinoa-BC and teff-BC media substitution also displayed additional amide I/II and ester-related absorptions, together with an additional peak around 1430 cm⁻^1^, indicating the presence of residual proteins and lipids, or a more amorphous matrix. These FTIR signatures likely reflect differences in feedstock nitrogen content, which is broadly predictive of amide and protein abundance in agro-derived substrates.

BC pellicles produced using triticale flour and modified HS control media yielded results similar to each other. This could be explained by earlier research which found that the FTIR characteristics of BC produced from wheat and rye generally align with those of BC produced from HS media (Güzel et al. 2025). As the physicochemical composition of triticale is closer to these generic cereals than to soy-BC, quinoa-BC or teff-BC, its alignment with the modified HS media results could probably be substantiated. The absence of lignin-associated absorption bands in all samples indicates that the cellulose produced was of high structural purity.

X-ray diffraction (XRD) analysis (Fig. 4b) validated the FTIR findings and revealed substrate-dependent differences in crystalline order. All BC samples retained the characteristic cellulose I reflections at 2θ = 14.5°, 16.5°, and 22.7°, corresponding to the (100), (010), and (200) planes of the Iα allomorph (Zhang et al. 2021). However, the crystallinity index (CrI %) varied substantially among treatments (Table 3). It was revealed that control BC exhibited the highest crystallinity (76%), indicative of densely packed microfibrils. BC produced from triticale-BC (65%) and soy-BC (64%) maintained similar values, preserving ordered cellulose I despite minor protein or lipid residues observed by FTIR. Meanwhile, teff-derived BC exhibited moderate crystallinity (42%), while quinoa-BC exhibited the lowest value (17%), consistent with weaker FTIR peaks and evidence of a less organized structure.

**Table 3:** The crystallinity index (CrI %) of the BC cellulose obtained from fermentation of modified HS and different variants of flour substrate, calculated by the Segal method based on background-subtracted XRD profiles

Sample	I₂₀₀ (22.7°)	I_am (18–19°)	Crystallinity Index (CrI, %)
Control	351	84	76.07
Soy-BC	316	115	63.61
Quinoa-BC	139	115	17.27
Triticale-BC	308	108	64.94
Teff-BC	108	62	42.49

By contrast, triticale and soy, the flours with the highest relative performance compared to the control (fold-change = 2.32 and 1.29, respectively), supported the formation of well-crystallized cellulose I with a denser fibrillar structure. In comparison, teff- and quinoa-derived BC (fold-change = 1.20 and 1.18, respectively) showed reduced crystallinity and looser microfibrillar structure.

### Thermogravimetric analysis (TGA)

**Fig. 5:**
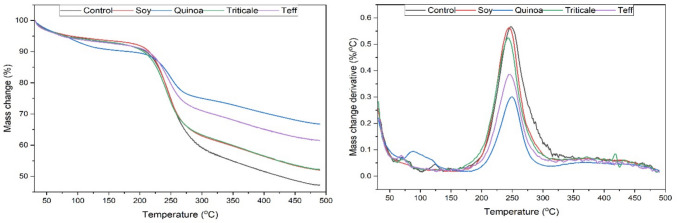
TGA and DTG curves of BC from five different samples substrates

Thermogravimetric (TGA) and derivative thermogravimetric (DTG) analyses were conducted to evaluate the thermal stability of BC produced from soy-BC, quinoa-BC, teff-BC, and triticale-BC media (Fig. 5). All samples exhibited the typical three-stage decomposition profile: initial moisture loss below 120 °C, major cellulose degradation between 220–350 °C, and gradual decline above 350 °C (Stumpf et al. 2013). The onset of significant mass loss occurred near 250 °C for most samples, corresponding to cellulose depolymerization, whereas quinoa-BC degraded earlier, reflecting lower structural stability (Ul-Islam et al. 2019). Among the cereal-derived variants, triticale-BC exhibited the greatest thermal resistance, retaining ~ 60% of its mass at 500 °C, consistent with its higher crystallinity and compact fibrillar architecture confirmed by XRD. Soy-BC showed a sharp DTG peak near 270 °C, comparable to triticale-BC and the control, although its broader profile suggested residual amide and ester groups detected by FTIR. Teff-BC demonstrated reduced stability, with an earlier onset of mass loss and broader, lower-intensity DTG peaks, consistent with its intermediate crystallinity (CrI = 48%) and SEM evidence of thicker, less ordered fibrils. By contrast, quinoa-BC displayed the lowest stability, with an attenuated DTG peak centered around 250 °C and higher char residue, consistent with its predominantly amorphous structure (CrI = 17%) and the disruptive influence of phytochemicals such as proteins and saponins (Zhang et al. 2021). Soy-, teff-, and triticale-derived BC remained stable below 240 °C, whereas quinoa-BC showed earlier mass loss near 100 °C from loosely bound water and volatile impurities (Ul-Islam et al. 2019). Above 380 °C, quinoa-BC retained higher residues, likely due to proteinaceous and microbial remnants (Zhang et al. 2021), while all samples exhibited maximum degradation temperatures within 240–250 °C.

## Discussion

The growth kinetics of *Komagataeibacter xylinus* DSMZ 2325 followed the typical pattern of a bacterial growth curve, comprising lag, exponential, and stationary phases (Rolfe et al. 2012). Cultures subsequently entered the exponential phase (24–54 h), characterized by balanced cell division and constant doubling of cell numbers (Al-Qadiri et al. 2008). The transition to stationary growth began at 48–50 h and coincided with the onset of BC pellicle formation, confirming that the late exponential to early stationary transition provides optimal conditions for cellulose biosynthesis (Reiniati et al. 2017). Compared with other bacteria, the exponential phase of *K. xylinus* was markedly prolonged. In contrast, *A. xylinum* 0416 initiates exponential growth within 10–15 h in HS medium (Fujikawa and Morozumi 2005), while *E. coli* typically exhibits shorter lag and log periods that are strongly temperature dependent: lag phases of 15–20 h at 10 °C, reduced to 1–2 h at 34 °C, with exponential phases lasting 6–8 h at higher temperatures and up to 20 h under cooler conditions (Fujikawa and Morozumi 2005). The extended exponential phase observed in the present study reflects the inherently slower metabolic rate and oxygen-dependent pathways characteristic of acetic acid bacteria. In the present study, growth plateaued from 72 h onward, marking the stationary phase when cell proliferation and death are balanced (Rolfe et al. 2012). This phase reflects nutrient depletion and metabolic stress (Sezonov et al. 2007) and is accompanied by a metabolic shift toward secondary metabolite formation. At this stage, BC synthesis is no longer sustained, as gluconic acid accumulates as a major byproduct, diverting carbon flux away from cellulose biosynthesis and reducing overall yields.

Nitrogen availability plays a decisive function in bacterial cellulose production by providing the building blocks for cell growth, enzyme synthesis, and polysaccharide biosynthesis (Carreira et al. 2011; Molina-Ramírez et al. 2017). The wide variation in nitrogen and protein content observed among the agricultural flours reflects their diverse biochemical composition and potential to sustain microbial metabolism. Soy flour, with the highest nitrogen content, supports robust BC formation due to its abundance amino acid and peptides content, which promotes enzyme activity and metabolic balance in *Komagataeibacter*. Interestingly, teff supported strong cellulose production despite its modest nitrogen level, suggesting that amino acid balance and mineral availability can compensate for limited total nitrogen. In contrast, spelt and millet, though richer in protein, produced comparatively lower yields, indicating that protein concentration solely does not predict considerably BC productivity. These findings highlight that nitrogen content defined by amino acid composition and nutrient accessibility could be as critical as total nitrogen concentration. This observation is consistent with prior reports on alternative nitrogen sources for BC fermentation, which emphasize that complex plant-derived substrates may influence cellulose yield through both nutritional and regulatory pathways (Molina-Ramírez et al. 2017).

The observed yield patterns demonstrate that both nitrogen quantity and protein quality influence BC productivity. Soy flour consistently supported superior yields due to its rich nitrogen reserve and balanced amino acid composition, which enhance enzyme synthesis and metabolic efficiency in *Komagataeibacter*. Quinoa and triticale also promoted high cellulose formation despite lower nitrogen content, suggesting that specific amino acid profiles or cofactor availability can compensate for reduced nitrogen input. Conversely, the poor performance of corn, rice, and sorghum media highlight that insufficient nitrogen limits cell proliferation and cellulose synthesis. However, beyond total nitrogen concentration, the bioavailability and composition of protein sources appear to be decisive under nitrogen-normalized conditions. These findings align with previous studies showing that amino acid balance, rather than solely crude protein content (Molina-Ramírez et al. 2017).

The screening assay confirmed that the BC yield is influenced by both the nitrogen value of the medium and the additional nutrient composition of the substrate. Under nitrogen-limited conditions (CNSM), overall yields decreased sharply, consistent with the dependence of *Komagataeibacter* on adequate nitrogen for enzyme synthesis and precursor formation (Molina-Ramírez et al. 2017). Soy maintained comparatively high productivity due to its high nitrogen content and balanced amino acid composition, while triticale and teff supported moderate yields despite lower nitrogen levels. In the CTN regime, where total nitrogen was constant, qualitative factors such as amino acid composition and the presence of minor nutrients became the key determinant of BC yield. The higher performance of triticale and teff suggests that their metabolic accessibility of nitrogen, likely due to favorable amino acid composition or the presence of cofactors that support *Komagataeibacter* metabolism. In contrast, the reduced yields observed for corn, bulgur, and rye imply that their nitrogen is less accessible, possibly linked to complex storage proteins or inhibitory phytochemicals that limit microbial utilization.

Medium pH also proved as a critical factor influencing BC yield. Initial pH values near the optimal range (5.5–5.8) promoted cellulose synthesis, while more acidic starting conditions reduced productivity. The convergence of all cultures to a final pH around 3.8 reflects gluconic acid accumulation, a characteristic byproduct of *Komagataeibacter* metabolism (Chawla et al. 2009; Bilgi et al. 2016). Maintaining a slightly acidic yet buffered pH therefore appears essential to sustain stable BC formation. These findings collectively highlight that both nitrogen speciation and initial medium pH exert strong, interdependent effects on BC productivity. Combining high-quality nitrogen sources with controlled pH adjustment provides a practical approach to enhance cellulose yield when using agro-derived substrates.

The findings of this study underscore the unique advantages of using cereal and pseudo-cereal flours as nitrogen-rich substrates for BC biosynthesis. Whereas most prior studies have focused primarily on carbon-source substitution, the systematic replacement of refined nitrogen sources has received comparatively limited attention, despite nitrogen contributing substantially to medium cost. By utilizing flours with well-defined nitrogen content, protein quality, and amino acid composition, this work introduces an alternative dimension of medium optimization, one that enables controlled evaluation of nitrogen availability and its direct influence on BC formation. Unlike heterogeneous agro-wastes, flours offer compositional reproducibility, allowing clearer mechanistic insight into how nitrogen-rich substrates shape both BC yield and material properties. Notably, several flour variants exceeded the performance of the modified HS control, illustrating their potential to simultaneously enhance productivity and reduce dependence on costly refined nitrogen inputs. These results broaden the current paradigm of BC bioprocess development by highlighting nitrogen-source innovation as a critical driver for improving yield, reducing production costs, and tailoring structural outcomes.

Key determinants of BC structure are strongly affected by both intrinsic and extrinsic factors such as producer strain, oxygen transfer, and medium composition (Volova et al. 2022). The SEM results confirmed that, even under equal nitrogen input, the chemical composition of the flour substrates had a pronounced effect on fibril organization and network integrity. Although individual fibril diameters varied only modestly, the relative abundance of micro- and nanofibrils shifted substantially, leading to distinct nanofibrillar architectures. The SEM imaging collectively demonstrates that even under constant nitrogen supply, substrate identity strongly influences BC ultrastructure, as shown in Fig. 3. Triticale-BC and teff-BC supported compact, integrated networks. The triticale and teff flour BC pellicles had compact, smooth and homogenous surface area, indicating that an ordered structure was formed. In contrast to these well-formed pellicle structures, quinoa-BC and soy-BC produced irregular and disrupted fibrils, likely influenced by phytochemicals such as saponins and isoflavones. These findings indicate that agro-derived flours are not only as cost-effective nitrogen sources but also as strong modulators of BC nanofibril assembly and network architecture. Quinoa’s saponins are known to inhibit diguanylate cyclase, lowering c-di-GMP levels and thereby reducing cellulose synthase activity (Ohana et al. 1998; Khan et al. 2018). Similarly, soy isoflavones may interfere with biofilm signaling and fibril crystallization, contributing to the observed disordered networks. According to the results obtained, pellicle nanostructure was not improved by using substrates with high nitrogen content. In general, more nutrient-rich substrates (triticale-BC and teff-BC) promoted compact, integrated fibril networks, whereas more phytochemical-rich flours (quinoa-BC and soy-BC) impaired fibril assembly, although direct evidence for these mechanisms in *Komagataeibacter* remains limited. The observed ability of different agro-based products to fine-tune fiber dimensions and network morphology through media supplementation could potentially be exploited as a toolkit for engineering BC with specific properties suited to various applications (Mohammadkazemi et al. 2015; Du et al. 2018; Wang et al. 2017).

The FTIR and XRD analyses corroborated the SEM observations, confirming that all BC samples preserved the cellulose I structure, characteristic of *Komagataeibacter*-derived cellulose. The spectra displayed the typical O–H, C–H, and C–O stretching vibrations, as well as CH₂ and C–H bending bands, consistent with previous reports for bacterial cellulose produced from conventional media (Revin et al. 2018; Reiniati et al. 2017; Henry et al. 2024). The absorption bands between 1160 and 1030 cm⁻^1^ and the β-(1 → 4)-glycosidic linkage signal at 895–900 cm⁻^1^ further confirmed the cellulose I allomorph (Irham et al. 2020; Avcioglu et al. 2021; Volova et al. 2022).

Additional amide and ester absorptions detected in soy-, quinoa-, and teff-derived BC suggest partial retention of nitrogen- and lipid-containing compounds from the respective feedstocks. These features indicate a more amorphous structure and reflect compositional differences among the substrates, particularly in nitrogen and protein content. In contrast, triticale-BC and the HS control exhibited almost identical FTIR profiles, implying that the chemical composition of triticale closely resembles that of conventional cereal-based media (Güzel et al. 2025). The absence of lignin-associated peaks in all spectra confirms the high purity of the cellulose produced and supports the efficiency of *K. xylinus* DSMZ 2325 in synthesizing uncontaminated cellulose matrices.

A previous study of Henry et al. 2018 reported that the FTIR spectra confirms that BC produced from agricultural waste (in treated SSF form) particularly maintains the essential chemical structure of pure cellulose, with minor variations that may offer opportunities for tailoring physicochemical properties. It is necessary to recognize that no characteristic lignin peaks were observed in the FTIR spectra, confirming the purity of the bacterial cellulose produced. This is consistent with the notable ability of our *K. xylinus* DSMZ 2325 strain to synthesise pure cellulose. The findings of the ATR-FTIR analysis also align with previous studies conducted using waste substrates such as rice bran and cereal dust for BC production (Abdelraof, et al. 2019; Castro, et al. 2011; Adebayo-Tayo et al. 2017) demonstrating the versatility of agro-based products as feedstock for BC synthesis (Fig. 4). The patterns obtained from FTIR analysis also suggested substrate-specific differences in fibril organization and crystallinity.

XRD analysis further clarified the influence of substrate composition on the crystalline organization of bacterial cellulose. All samples exhibited the characteristic cellulose I reflections at 2θ = 14.5°, 16.5°, and 22.7°, yet their crystallinity indices varied significantly. Triticale- and soy-derived BC displayed the highest crystallinity (65% and 64%), consistent with the compact fibril arrangements observed microscopically. Quinoa-derived BC exhibited the lowest crystallinity (17%), consistent with attenuated FTIR bands and enriched protein-associated absorptions. By contrast, triticale and soy, the flours with the highest relative performance compared to the control (fold-change = 2.32 and 1.29, respectively), supported the formation of crystalline cellulose I with a denser fibrillar structure. In comparison, teff- and quinoa-derived BC (fold-change = 1.20 and 1.18, respectively) showed reduced crystallinity and looser network organization.

Analysis of these results indicates that, similar to FTIR characteristics, the crystallinity values obtained from XRD did not directly correlate with feedstock nitrogen content. Instead, the data confirm earlier reports that higher crystallinity corresponds to sharper XRD peaks and improved mechanical properties, including greater tensile strength (Molina-Ramírez et al. 2017; Henry et al. 2024), as observed in the denser pellicles from triticale- and soy-based media. Conversely, the results also align with studies linking lower crystallinity to increased water uptake and swelling capacity (Xu et al. 2022), consistent with the less compact pellicles from teff and quinoa substrates.

In summary, FTIR and XRD analyses confirmed the preservation of cellulose I structures in BC obtained from all four flour substrates. Some samples displayed additional peaks, suggesting subtle modifications to surface chemistry or crystallinity. These minor alterations may enhance properties such as thermal stability or reactivity without compromising the essential cellulose structure. Overall, the findings indicate that flour substrate identity not only modulates fibril assembly but also provides a route to tailor BC properties through agricultural substrate selection and nitrogen supplementation strategies.

The results of the TGA and DTG analyses correlate closely with the results of the XRD experiments but are not entirely coherent with observations from the SEM and FTIR analyses. While characteristics such as fibril diameter and residue content could predict structural stability in some cases, it is ultimately the reduction of XRD crystallinity which correlated to the reduced mechanical properties of a pellicle, and to its vulnerability to decomposition. This can be well demonstrated in the case of teff-BC which was shown to be less stable than soy-BC in correlation with its lower crystallinity, despite having a relatively well-formed structure and the widest fibrils from among all the samples according to SEM imaging. Even though soy-BC was observed to have inferior structure and lower fibril diameter, its higher fibril density provided higher crystallinity, and therefore better outcomes during the TGA and DTG experiments.

## Conclusion

Agro-derived flours represent effective substitutes for yeast extract and tryptone in BC production, with performance influenced not only by nitrogen availability but also by nutrient composition and phytochemical content. Under nitrogen-normalized conditions, triticale, teff, quinoa, and soy supported significant yield increases, whereas under nitrogen-limited conditions, soy performed best, together with triticale, teff, and sorghum.

These results demonstrate that higher nitrogen content in the medium did not consistently correlate with increased yields, and that factors such as pH and the presence of specific nutrients or phytochemicals can be more decisive in determining BC productivity. Structural and thermal analyses further showed that nutrient-rich substrates preserved fibril order, while phytochemical-rich substrates introduced amorphous domains that disrupted nanocellulose assembly. Crystallinity emerged as the most significant structural parameter, as it correlated more strongly with thermal stability and pellicle integrity than fibril order or diameter.

Overall, the experiments highlight that the structural and physicochemical characteristics of BC depend strongly on the type of agro-based substrate used, thereby enabling modulation of its key properties. This provides a basis for the targeted development of BC products tailored for specialized applications.

Future studies should further investigate the effects of controlled variations in nitrogen supply, pH, nutrient balance, and phytochemical composition to better understand how agro-derived products modulate BC biosynthesis. As the overarching aim is to identify sustainable alternatives to conventional media, future evaluations should also consider the economic feasibility and environmental impact of different agro-based supplements.

## Data Availability

All data generated or analyzed during this study are included in this published article and its supplementary information files.
